# Ulcerated Tophaceous Gout

**DOI:** 10.7759/cureus.28729

**Published:** 2022-09-03

**Authors:** Hovra Zahoor, Ronak Patel, Jessica El-bahri

**Affiliations:** 1 Internal Medicine, HCA Orange Park Hospital, Orange Park, USA; 2 Radiology, Baylor College of Medicine, Houston, USA

**Keywords:** management of ulcerated tophi, gout crystals, ulcerated tophaceous gout, ulcerated tophi, tophaceous gout

## Abstract

Gout is a disease that occurs in response to the presence of monosodium urate (MSU) crystals typically within joints. Patients with gout may develop a chronic deposition of monosodium urate crystals within or around joints, cartilage, tendons, peri-articular, and subcutaneous tissue. This condition is termed “tophaceous gout.” Ulceration of the skin by tophi is very uncommon. Literature regarding the clinical course and the management of ulcerated tophi is limited and, therefore, treatment options are not well established. We hereby present a case of a 46-year-old male who presented to our facility with poorly controlled polyarticular tophaceous gout complicated by ulcerated tophi. Our hope is to contribute to the limited knowledge of this rare disease process and to contribute toward formulating the best management approach.

## Introduction

Gout (monosodium urate crystal deposition disease) is described biochemically by extracellular fluid urate saturation, reflecting as hyperuricemia, with serum or plasma urate concentrations greater than 6.8 mg/dL. The clinical manifestation of gout is variable and may include chronic arthropathy, intermittent flares of inflammatory arthritis, tophaceous gout, or uric acid nephrolithiasis. Approximately 12-35% of all patients with gout develop tophaceous gout [1-4]. Tophaceous gout is characterized by collections of solid urate accompanied by chronic inflammatory and often destructive changes in the surrounding connective tissue. Patients with tophaceous gout have been known to have a poor quality of life, increased healthcare resource use, and significant morbidity [1]. Despite the high prevalence of tophaceous gout, ulceration is surprisingly uncommon and is an exception rather than the rule.

## Case presentation

A 46-year-old male with a past medical history of gout, chronic kidney disease stage 2, and hypertension presented with complaints of right knee erythema, drainage, and dull pain. The patient reported being diagnosed with gout approximately 10 years ago but had been self-treating gout with cherry juice. He reported no active gout flare during this time period but did endorse multiple tophi in various joints. He reported occasional dull pain, for which he took ibuprofen as needed.

The patient stated that he bumped his knee approximately one to two months ago and had a small cut on the right knee tophus. At that time, he was seen at urgent care where topical and oral antibiotics were prescribed, which improved his symptoms. However, in the week prior to presentation, his right knee exhibited increased erythema and swelling complicated by the tophus opening up with bleeding and drainage of white chalky material. This prompted him to present to the local emergency department where he was given vancomycin and piperacillin-tazobactam. He did not have any associated symptoms of fever, chills, or any other joint pain at that time. X-ray was performed, which revealed no knee joint involvement. He was then transferred to our facility.

**Figure 1:**
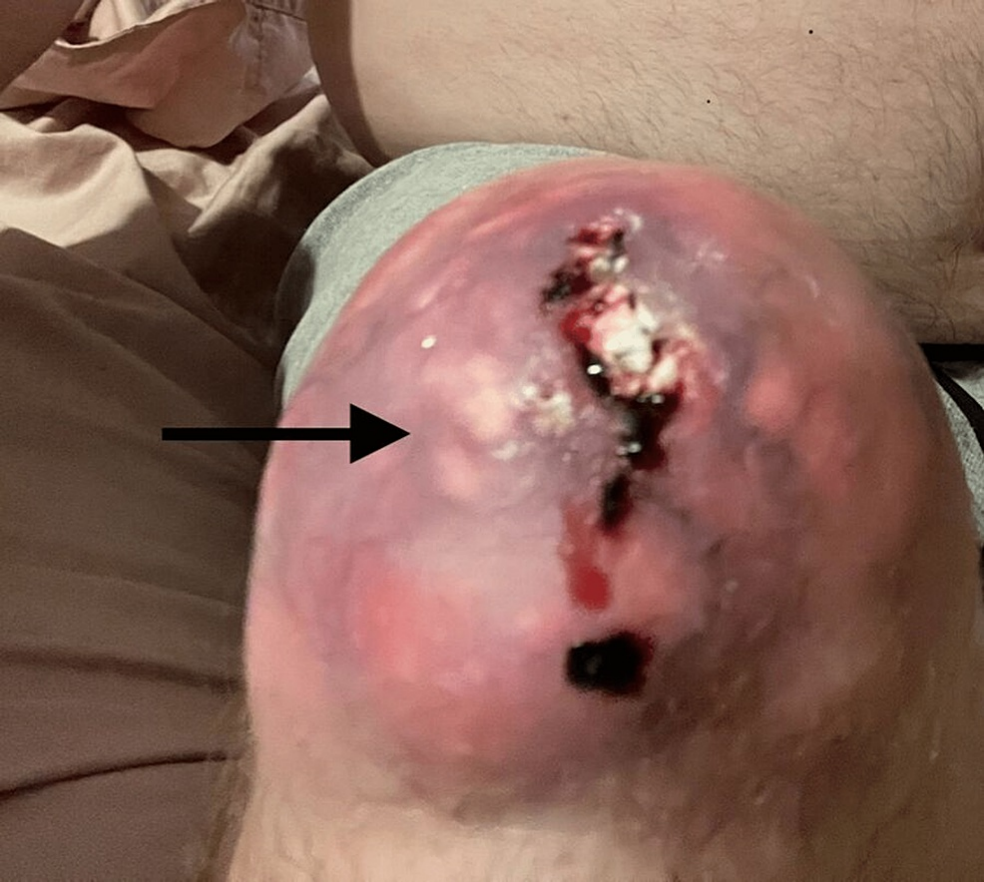
Right knee ulcerated tophus

**Figure 2:**
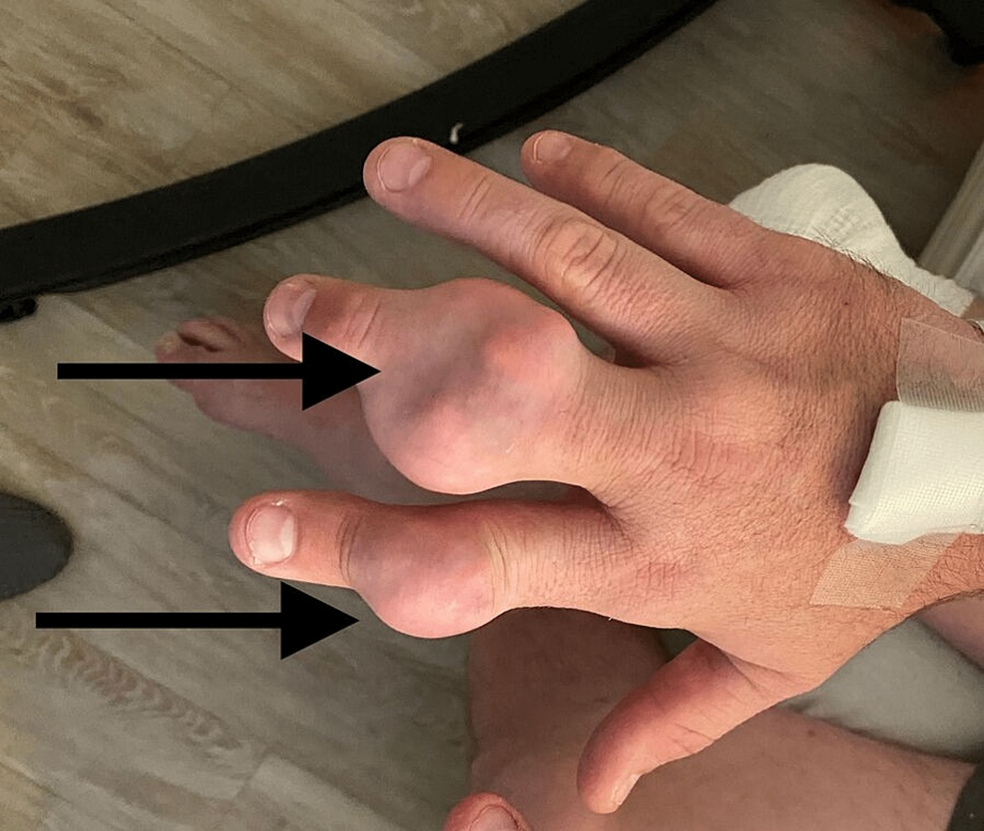
Right second and third digit interphalangeal joint tophi

**Figure 3:**
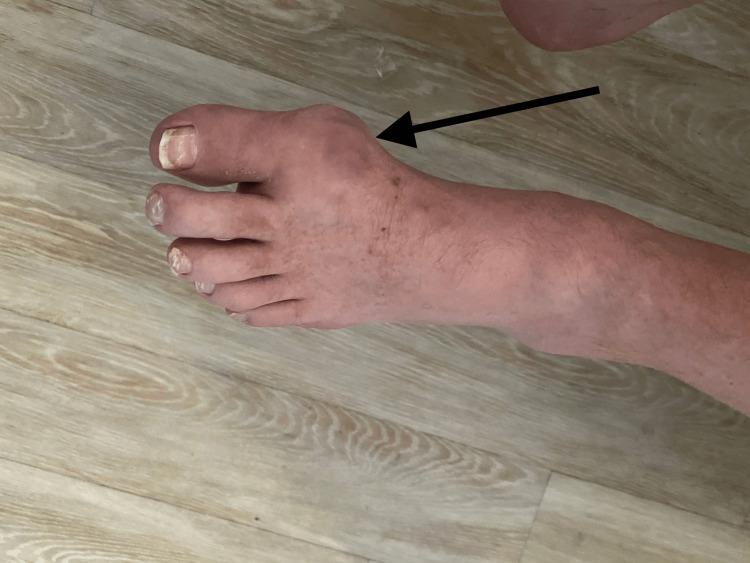
Left first metatarsophalangeal joint tophus

**Figure 4:**
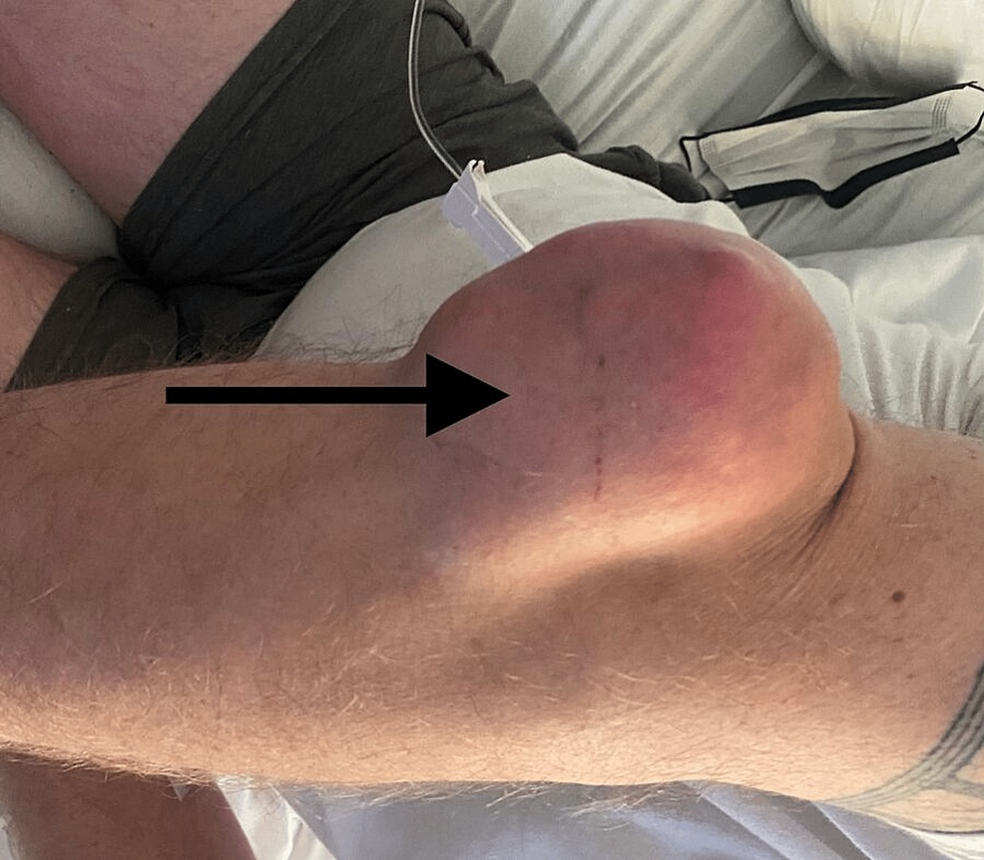
Right elbow joint tophus

On the physical examination, his vital signs were stable. Musculoskeletal examination revealed an erythematous and swollen right knee with active drainage of blood and white chalky material (Figure 1). Additional findings included tophi on the right knee, right and left elbows, distal interphalangeal joint of the right index finger and middle finger, interphalangeal joint of the left thumb, and right and left toe metatarsophalangeal joints (Figures 2-4).

 

**Figure 5:**
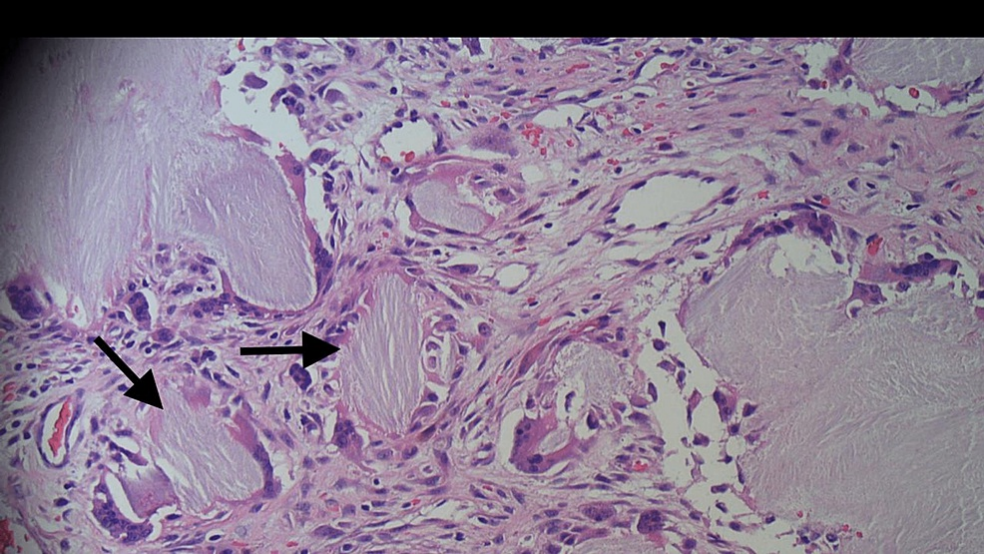
Histopathology revealing fibrinous amorphous material deposits and collection of crystals (rod and needle shaped crystals depicted by arrows)

On further workup, basic labs were unremarkable except for creatinine of 1.17 mg/dL and uric acid level of 8.4 mg/dL. For management, the patient was started on prednisone 50mg, colchicine 0.6mg, and allopurinol 100mg. The patient was switched to intravenous vancomycin and ceftriaxone. Wound care service was consulted and appropriate care was provided. After a thorough discussion, dermatology and orthopedic surgery services were consulted and the patient was evaluated. The dermatology service performed a skin biopsy, which revealed unremarkable benign appearing epithelial cells. Orthopedic surgery service performed surgical excision of the right knee tophus. Excised contents were sent for histopathological examination. The histopathology revealed a well-circumscribed lobulated mass in association with fibrinous amorphous material deposits and collection of crystals (rod and needle shaped crystals) (Figure 5), which were consistent with uric acid crystals (gouty tophi). There was no evidence of malignancy, but reactive giant cell granulomatous chronic inflammation was noted.

Post-operatively, the patient remained stable. Intraoperative wound culture, however, revealed *Pseudomonas aeruginosa*. Antibiotic regimen was adjusted to include cefepime while sensitivities were awaited. The final report revealed sensitivity to ciprofloxacin, and the patient was switched to oral ciprofloxacin. The patient was subsequently discharged to short-term rehabilitation with recommendations to follow up with his primary care physician, orthopedics, and rheumatology as an outpatient.

The patient subsequently followed up with us in the outpatient clinic. He reported that he was doing well and was able to perform his daily activities. He reported following up with physical therapy and taking medications as prescribed. He continued to follow up with us in the outpatient clinic. His clinical course remained uncomplicated.

## Discussion

Affecting greater than 8 million Americans, gout is the most common inflammatory arthropathy [5]. It is caused by the deposition of monosodium urate crystals in the synovial fluid and surrounding tissues and is most commonly associated with serum uric acid levels > 6.8 mg/dL. However, approximately 30% of patients with gout and inadequate control of hyperuricemia develop tophaceous gout, which is a subtype of disease characterized by chronic deposition of crystals [6]. This deposition is typically known to occur within or around joints and is called tophi. Tophi, however, can also develop in other areas such as tendons, subcutaneous tissue creating bulae, heart valves, and cornea [7]. Ulceration of tophi is uncommmon; therefore, limited data are available about its clinical course and management [8].

A recent literature review examined cases of ulcerated tophaceous gout and found nine articles comprising 22 individual patient cases. The age of the affected patient population ranged from 36 to 95 years. Men (82%) were more commonly affected, and comorbidities including diabetes and peripheral vascular disease were found in most of the patients. The greatest prevalence of the 43 total ulcers occurred on the feet [8]. It is to be noted that our patient lacked the classic clinical presentation that was seen in previously reported cases. He endorsed an ulcer in the tophi in the right knee and did not have the commonly associated comorbidities including diabetes or peripheral vascular disease, as noted in the literature review.

The literature regarding the management of ulcerated tophi is limited, and there are no established treatment modalities for ulcerated tophi. The treatment approaches that have been attempted include topical treatments such as the use of 3% citric acid in petroleum jelly topically and a variety of debridement methods. These debridement techniques include single debridement of the tophus with or without preceding topical treatments, using a hydrogel to soften the tophus followed by gentle monthly debridement and free flap surgery to cover the wound. These treatment modalities yielded promising results, and the time to healing ranged from 7 to 40 days [4]. However, limited data are available on the operative correction of tophaceous gout. There is lack of statistically robust data on specific outcome measures with operative correction of tophaceous gout. The lack of controlled clinical studies makes it a challenge to determine when, or if, a specific patient should be referred for surgery to remove tophi.

Our patient endorsed debilitating tophi in the right knee with ulcer formation. He was functional at baseline and worked actively despite multiple tophi until the recent ulceration of the tophi in the right knee. Various treatment options were discussed based on the limited data available. Given the patient’s good baseline health and functional status, as well as lack of comorbidities that would impair wound healing, we decided to proceed with the surgical excision of the tophus. Our patient had a successful surgical removal of right knee ulcerated tophus and a successful post-operative course.

## Conclusions

Limited data are available on the demographics, risk factors, and treatment options for patients with ulcerated tophi. Our case highlights the surgical excision of ulcerated tophus as a viable treatment modality for patients with ulcerated tophaceous gout, which had not been attempted in previously reported cases. We hope to bring attention to the need for further research in this direction with the goal of formulating the best medical, surgical, and wound care regimens for the treatment of tophaceous ulcers.
